# Supplementary material for: Ambient PM2.5 exposure and expected premature mortality to 2100 in India under climate change scenarios
Source: Nat Commun. 2018 Jan 22;9:318. doi: 10.1038/s41467-017-02755-y (PMC5778135; doi:10.1038/s41467-017-02755-y)
Supplement: Supplementary file 1 — Supplementary Information [file 41467_2017_2755_MOESM1_ESM.pdf]

Supplementary Note

Shared Socioeconomic Pathways scenarios

In order to assess the relationships between socio-economic development in response to climate change, the Integrated Assessment Modelling (IAM) and the Impacts, Adaptation and Vulnerability (IAV) have launched five shared socioeconomic pathways (SSPs) [1,2] that describe the future world with respect to social and economic mitigation and adaptation challenges. This new set of scenarios provide projections by age, sex and six levels of education for all the countries[3], and they can be used to explore the costs and benefits of climate policy and assess different aspects and effects of climate change[4]. The five SSP scenarios are a green growth strategy (SSP1), a middle of the road development pattern (SSP2), a fragmentation between the regions (SSP3), an increase in inequality across and within regions (SSP4) and a fossil-fuel based economic development (SSP5). The population distributions that were used as drivers to develop these scenarios are used in our study. The detailed description of each of the SSP scenarios is provided in a recent article[3]. Here we explain each scenario briefly.

In SSP1 (sustainability) scenario, the world is expected to make a good progress towards sustainability with ongoing efforts to achieve development goals while reducing resource intensity and fossil-fuel dependency. The population component in the SSP 1 is known as 'rapid development'. This scenario assumes that education and health investments accelerate the demographic transition, leading to a relatively low population. The countries are grouped into 3 sections, high fertility, low fertility and rich OECD (organization for economic co-operation and development). Low mortality and high education was assumed for all the 3 groups, whereas medium fertility scenario was chosen for the rich OECD countries and low fertility scenario was chosen for the other two groups. Migration level was assumed to be medium for all the countries.

In SSP2 (middle of the Road) scenario, trends typical to recent decades continue with some progress towards achieving development goals, historic reductions in resource and energy and slowly decreasing fossil fuel dependency. The population component of this scenario is described as ‘medium’. Fertility, mortality and migration are assumed to be 'medium' for all the 3 groups of countries.

SSP3 (fragmentation) scenario is narrated in such a way that it is opposite to sustainability. The population of this scenario is described as ‘stalled development’, which describes a world with stalled demographic transition. This scenario assumes high mortality and low education in all the three groups of countries. Fertility is assumed to be low in the rich OECD group and high in the other two groups, and migration is assumed to be low for all the countries.

SSP4 (inequality) scenario predicts a very unequal world both within and across the countries. The population component of SS4 is described as ‘inequality’. Mortality and fertility are considered to be high in the high fertility countries. The low fertility countries are assumed to suffer from medium levels of mortality.

SSP5 (conventional development) scenario envisions a world that stresses conventional development oriented towards economic growth. The population component has the same name as the scenario itself. The world features high educational attainment and low mortality, whereas high fertility is assumed for rich OECD countries and low for all other countries.

Representative Concentration Pathways scenarios

The RCPs[5,6] are a set of four new pathways developed for the climate modelling community as a basis for long-term and near-term modelling experiments. The four RCPs represent the projected top-of-the-atmosphere radiative forcing stabilized at 2.6, 4.5, 6 and 8.5 W/m^2 (hence denoted as RCP2.6, RCP4.5, RCP6 and RCP8.5) at the end of 21st century. The RCPs are developed by collaboration between integrated assessment modellers, climate modellers,

terrestrial ecosystem modellers and emission inventory experts. Land use and emissions of air pollutants and greenhouse gases are reported mostly at a 0.5º×0.5º spatial resolution, with air pollutants also provided per sector (for well-mixed gases, a coarser resolution is used). The storylines of these RCPs were utilized by the CMIP5 climate modelling group to predict concentration of air pollutants and meteorological factors for the future. In this study we use projections of the CMIP5 models under 2 RCP scenarios - RCP4.5 and RCP8.5.

RCP 4.5[7] scenario is developed by the MiniCAM modelling team at the Pacific Northwest National Laboratory's Joint Global Change Research Institute (JGCRI). It is a stabilization scenario where total radiative forcing is stabilized before 2100 at 4.5 W/m^2 without ever exceeding that value (approximately 650ppm CO_2 equivalent.) by employment of a range of technologies and strategies for reducing greenhouse gas emissions. The RCP8.5[8] scenario was developed by the group at International Institute for Applied Systems Analysis (IIASA), it combines assumptions about high population and relatively slow income growth with modest rates of technological change and energy intensity improvements, leading in the long term to high energy demand and GHG emissions in absence of climate change policies. Compared to the other RCP scenarios, RCP8.5 thus corresponds to the pathway with the highest greenhouse gas emissions. The greenhouse gas emission and concentration in this scenario increase over time to lead to a radiative forcing of about 8.5 W/m^2 (1370 CO_2 equivalent) by the end of the century.

Supplementary Table 1: List of CMIP5 models along with their spatial resolution (in degrees); output of which are analysed to project ambient $PM_{2.5}$ concentration.

Model Name	Model Spatial Resolution (in degrees)
HadGEM2-CC	1.25 × 1.875
HadGEM2-ES	1.25 × 1.875
IPSL-CM5A-LR	1.875 × 3.75
IPSL-CM5B-LR	1.875 x 3.75
MIROC 5	1.4065 × 1.4065
MIROC ESM	2.8125 × 2.8125
MIROC ESM Chem	2.8125 × 2.8125
MRI CGCM3	1.125 × 1.125
NorESM1-M	1.875 × 2.5
NorESM1-ME	1.875 × 2.5
GFDL CM3	2 × 2.5
GFDL ESM2M	2 × 2.5
GFDL ESM2G	2 × 2.5

Supplementary Table 2: Projected mean estimates (±uncertainty) of decadal premature mortality burden per 100,000 exposed population from ambient $PM_{2.5}$ exposure for RCP4.5 and RCP8.5 scenarios. Total premature deaths for decades 2031-2040 (near future), 2061-2070 (distant future) and 2091-2100 (far future) are shown in Table 1.

	SSP1		SSP2		SSP3		SSP4		SSP5	
**Decade**	**RCP4.5**	**RCP8.5**	**RCP4.5**	**RCP8.5**	**RCP4.5**	**RCP8.5**	**RCP4.5**	**RCP8.5**	**RCP4.5**	**RCP8.5**
**2011-2020**	29.7 (±9.6)	30.5(±9.8)#	30.4(±9.9)	31.2(±10.1)	30.9(±10.1)	31.7(±10.3)	30.8(±10.1)	31.6(±10.2)	29.6(±9.6)	30.3(±9.8)
**2021-2030**	20.8(±6.6)	21.3(±6.8)#	22.5(±7.2)	23.1(±7.4)	24.4(±7.9)	24.4(±8.1)	23.7(±7.6)	24.3(±7.8)	20.1(±6.4)	20.6(±6.5)
**2031-2040**	16.1(±5.1)	16.6(±5.2)#	18.3(±5.8)	18.9(±5.9)	21.4(±6.9)	22.2(±7.1)	20.0(±6.4)	20.7(±6.5)	14.8(±4.6)	15.3(±4.7)
**2041-2050**	12.7(±3.9)	13.5(±4.1)#	15.2(±4.8)	16.1(±5.0)	19.5(±6.3)	20.7(±6.6)	17.2(±5.5)	18.3(±5.7)	11.3(±3.5)	12.0(±3.7)
**2051-2060**	10.1(±3.2)	11.1(±3.4)#	12.4(4.0)	13.7(±4.3)	17.7(±5.8)	19.6(±6.3)	14.5(±4.7)	16.1(±5.1)	8.7(±2.7)	9.6(±2.9)
**2061-2070**	7.9(±2.6)	9.4(±2.9)#	10.1(±3.3)	11.8(±3.7)	15.8(±5.3)	18.5(±5.9)	12.1(±4.0)	14.1(±4.5)	6.6(±2.1)	7.8(±2.4)
**2071-2080**	6.2(±2.2)	8.1(±2.5)#	8.0(±2.8)	10.3(±3.2)	13.8(±4.9)	17.8(±5.7)	9.8(±3.5)	12.6(±4.0)	4.9(±1.7)	6.4(±1.9)
**2081-2090**	4.9(±1.9)	6.8(±2.1)#	6.6(±2.5)	8.9(±2.8)	12.4(±4.8)	16.8(±5.4)	8.2(3.2)	11.1(±3.5)	3.8(±1.5)	5.3(±1.6)
**2091-2100**	4.3(±1.7)	5.8(±1.8)#	5.8(±2.4)	7.7(±2.5)	11.8(±4.8)	15.6(±5.2)	7.3(±2.9)	9.6(±3.1)	3.2(±1.3)	4.3(±1.4)

#Combination of RCP8.5 scenario and SSP1 population is practically impossible.

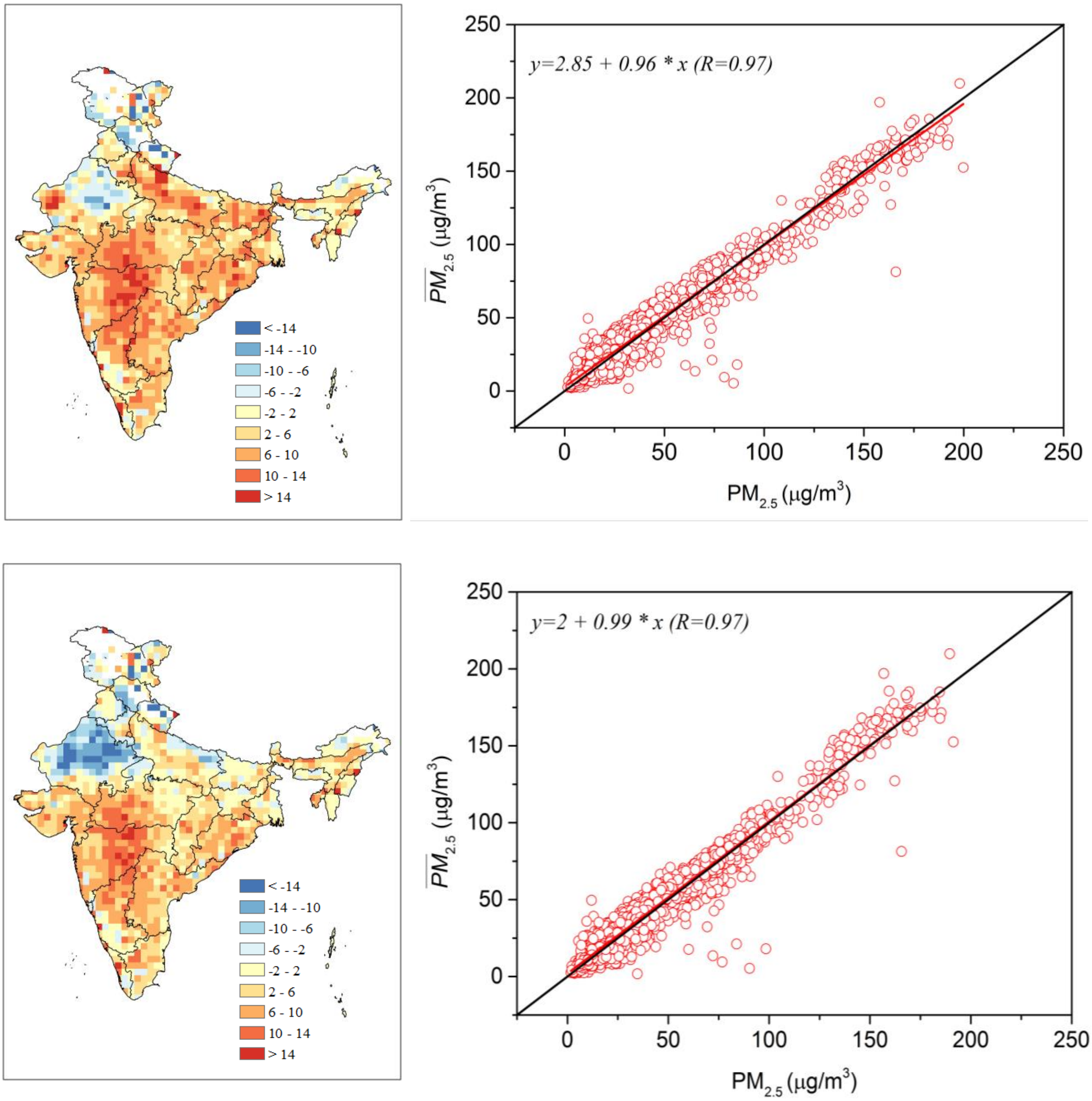

Supplementary Figure 1. (Left panel) Spatial distribution of bias and (right panel) linear regression statistics from comparison of CMIP5 model-derived $PM_{2.5}$ concentration against MISR-retrieved $PM_{2.5}$ in (top) RCP4.5 and (bottom) RCP8.5 scenario for the period 2011-2015. The figures in the right panel compares model derived $PM_{2.5}$ (y-axis) and satellite derived $PM_{2.5}$ (x-axis). Each red dot represents average $PM_{2.5}$ exposure for each grid cell in the figures on the left panel. The black box encompasses the desert region in Rajasthan where the CMIP5 models overestimate $PM_{2.5}$ exposure relative to MISR-derived $PM_{2.5}$

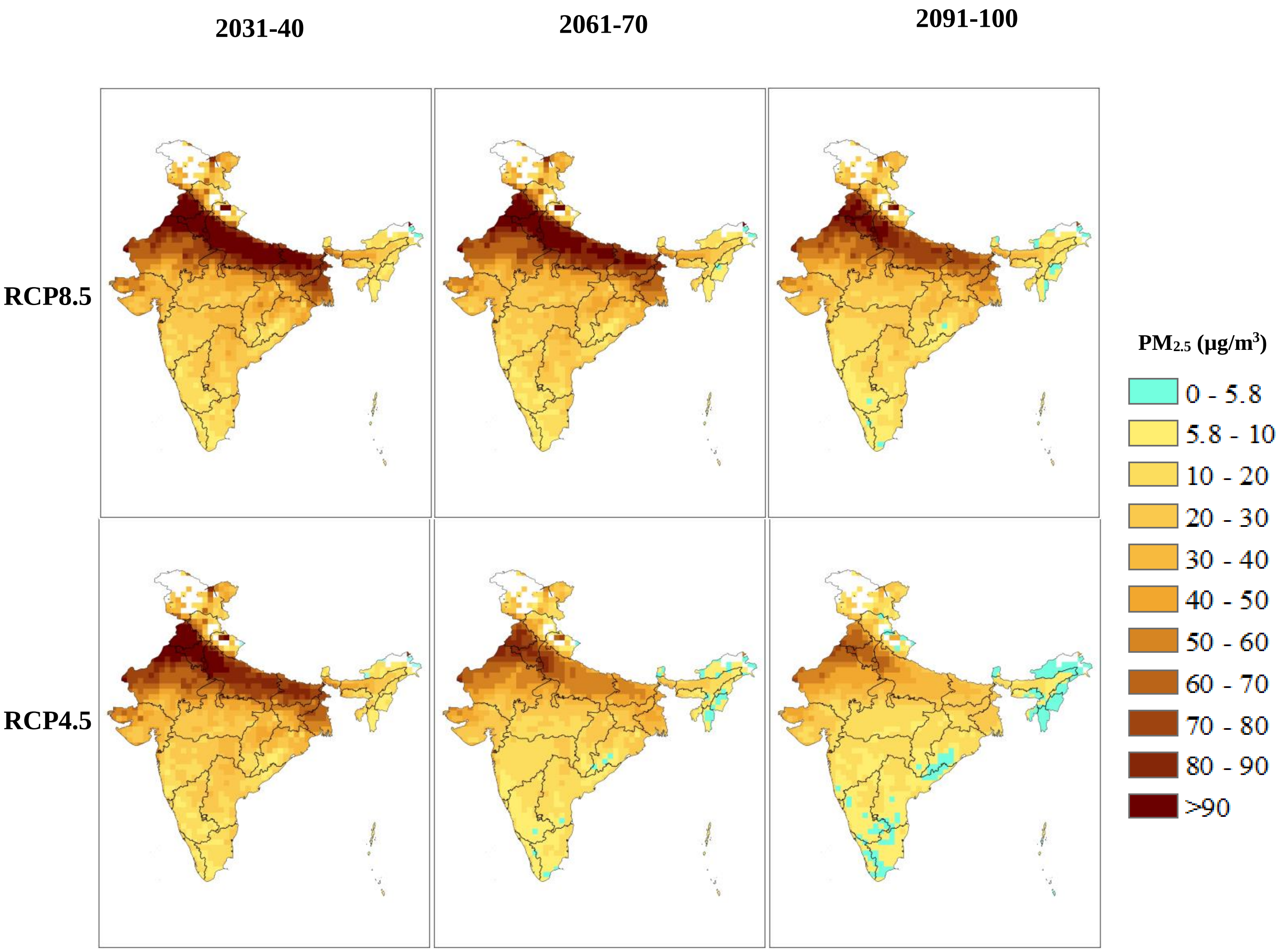

Supplementary Figure 2. Spatial patterns of ambient $PM_{2.5}$ concentration (µg/m^3) in three representative decades of (left) near future (2031-2040), (middle) distant future (2061-2070) and (right) far future (2091-2100) under (top panel) RCP8.5 and (bottom panel) RCP4.5 scenario.

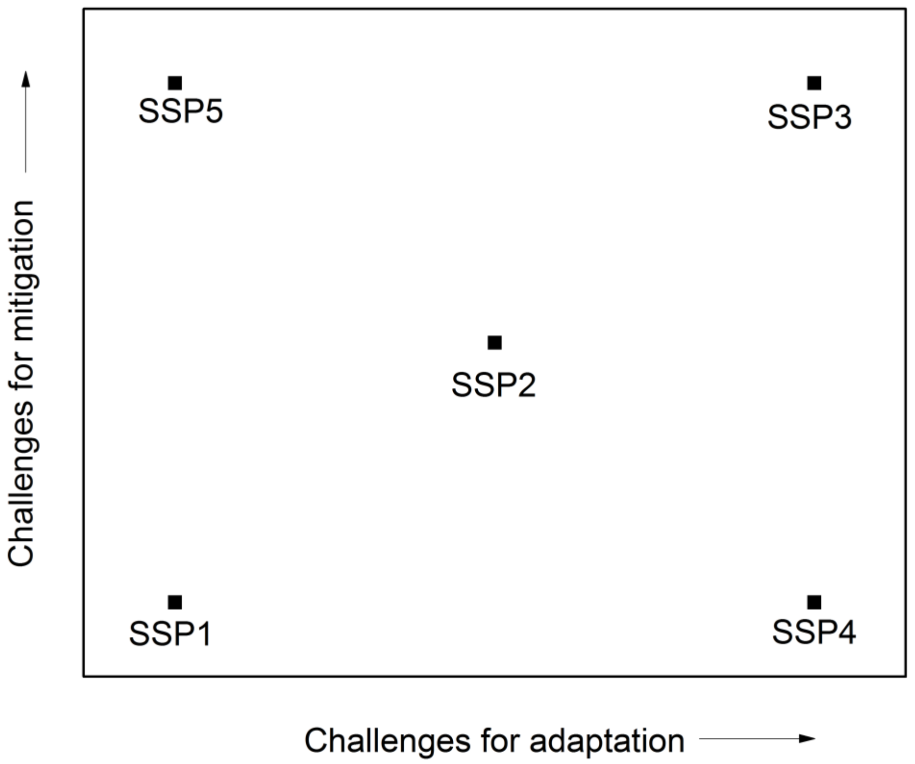

Supplementary Figure 3. Depiction of the challenges for mitigation and adaptation (in relative scale) for the 5 SSP scenarios.

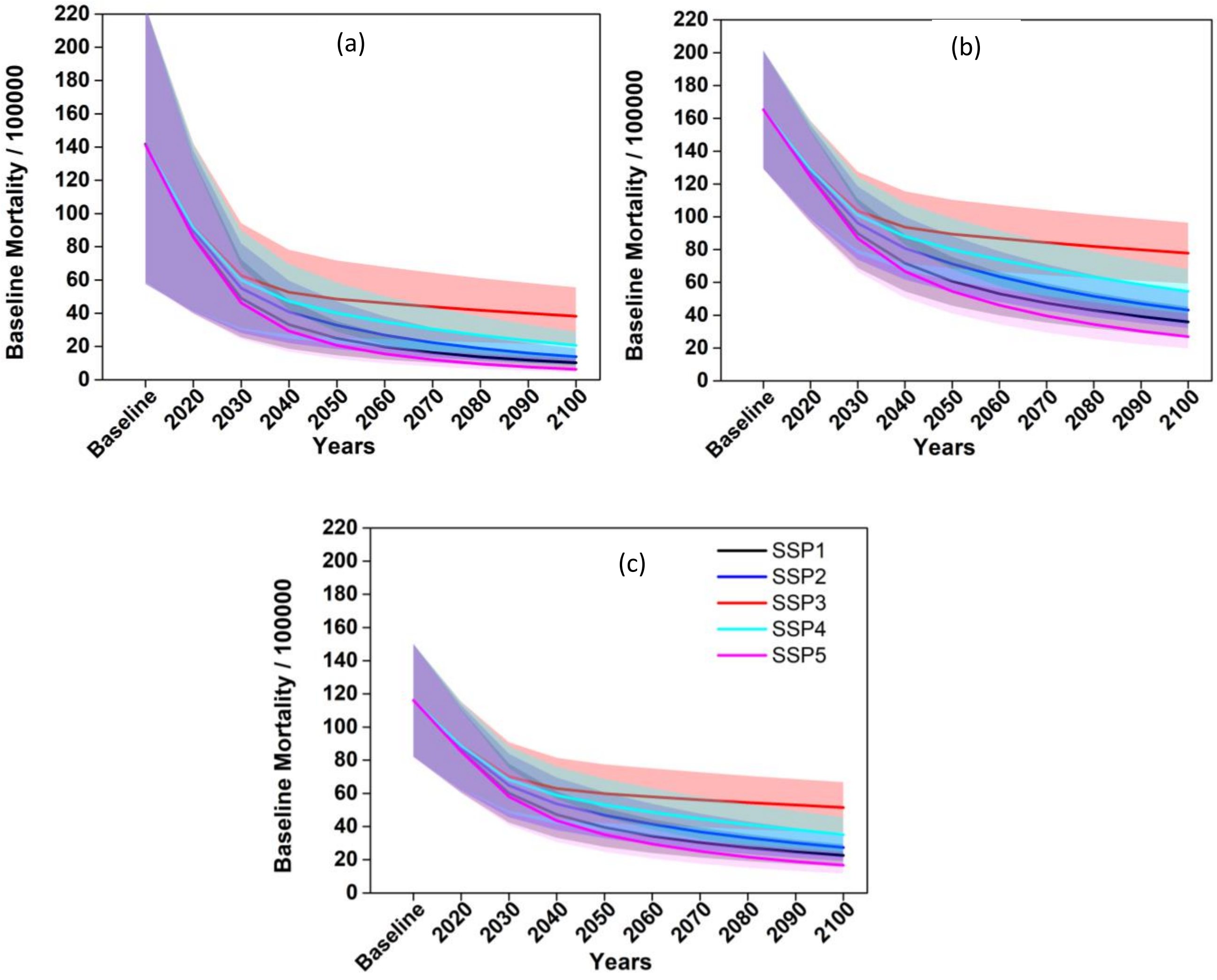

Supplementary Figure 4. Projected changes in baseline mortality (per 100,000 population) for (a) COPD, (b) IHD and (c) stroke in five SSP scenarios using the present-day relation between baseline mortality and GDP[9]. The shaded region in the plots represent the uncertainty range in baseline mortality estimated using the ranges in the coefficients of the non-linear GDP-baseline mortality functions as in *(http://www.sciencedirect.com/science/article/pii/S0160412016300848)*

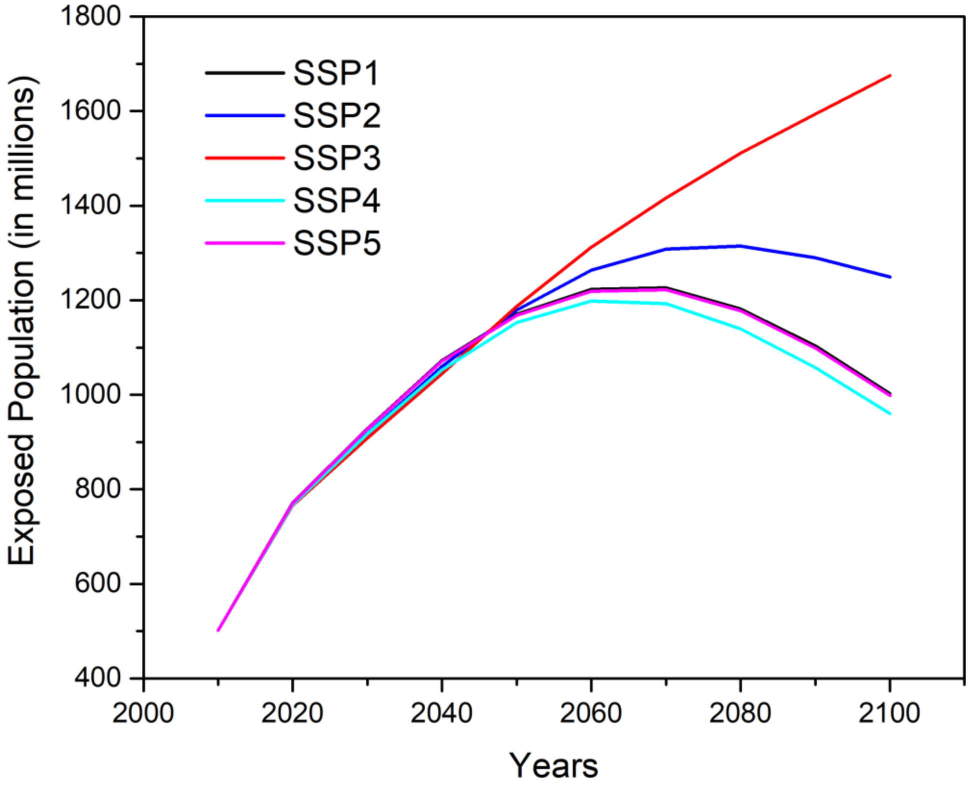

Supplementary Figure 5. Projected changes in exposed population (above 25 years) for each of the five SSP scenario populations in India.

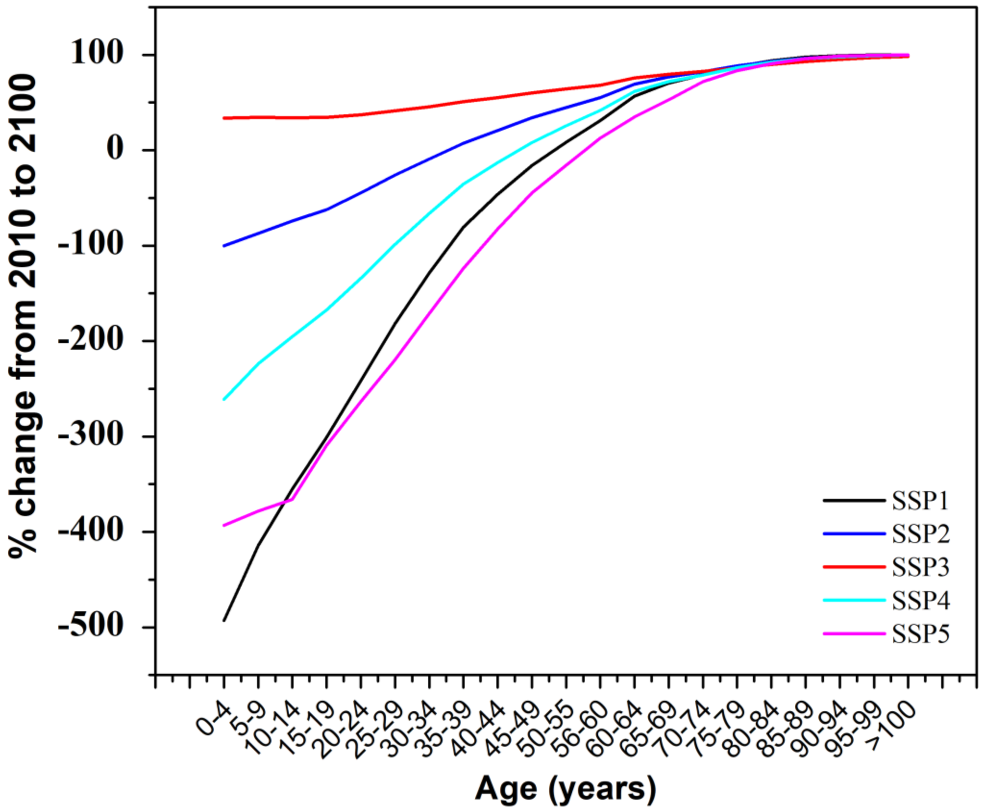

Supplementary Figure 6. Projected changes in demographic patterns of 2100 relative to 2010 for the 5 SSP scenario populations

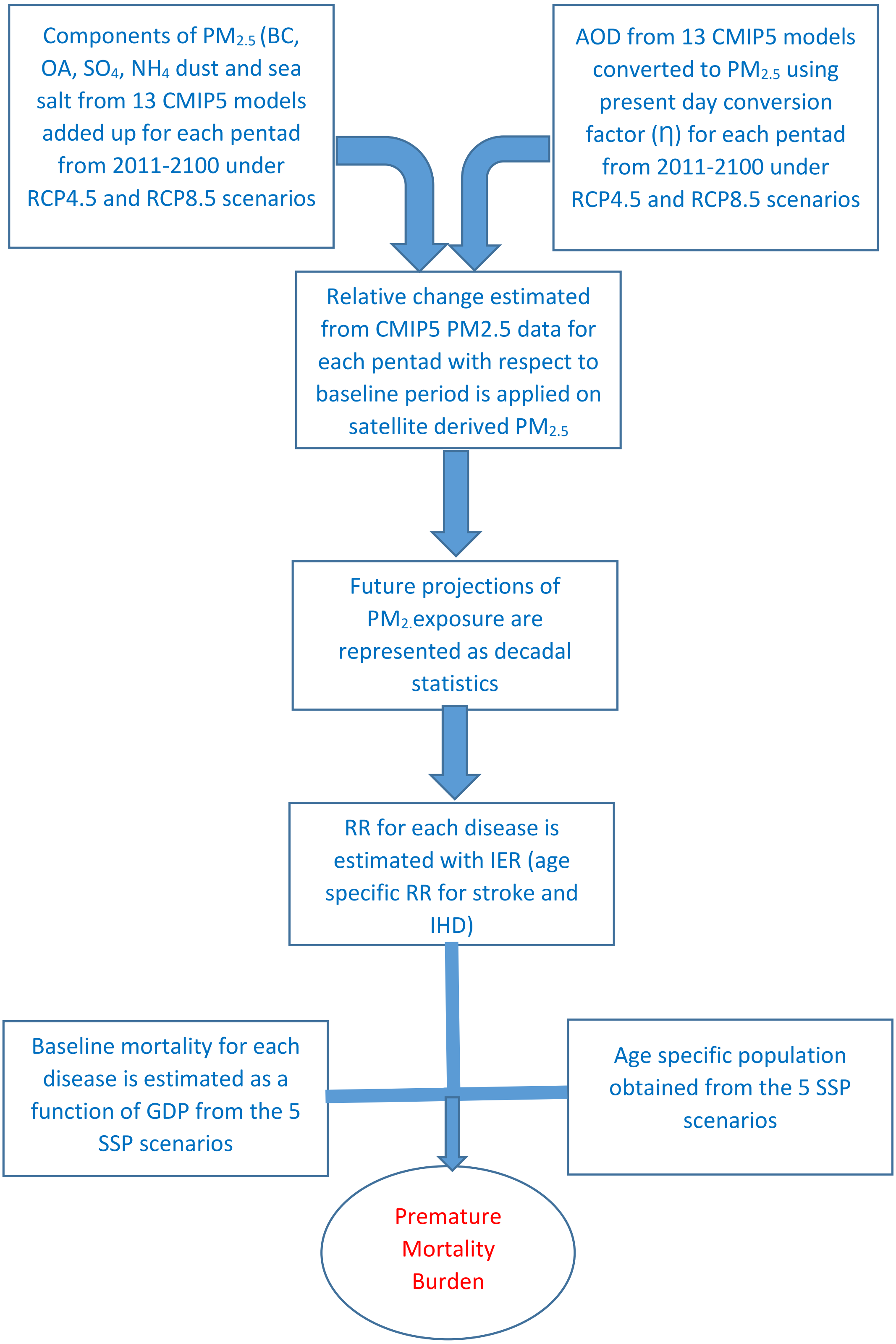

Supplementary Figure 7: Schematic description of the methodology used in this study.

Supplementary References

1. O'Neill, B. C. *et al.* Workshop on The Nature and Use of New Socioeconomic Pathways for Climate Change Research Core Writing Team Acknowledgments. *Meet. Rep. Work. Nat. Use New Socioecon. Pathways Clim. Chang. Res.* 1–37 (2012). at <http://www.isp.ucar.edu/socio-economic-pathways>
2. Ebi, K. L. *et al.* A new scenario framework for climate change research : background , process , and future directions. 363–372 (2014). doi:10.1007/s10584-013-0912-3
3. Kc, S. & Lutz, W. The human core of the shared socioeconomic pathways : Population scenarios by age , sex and level of education for all countries to 2100. *Glob. Environ. Chang.* (2014). doi:10.1016/j.gloenvcha.2014.06.004
4. Vuuren, D. P. Van, Kriegler, E. & Neill, B. C. O. A new scenario framework for Climate Change Research : scenario matrix architecture. 373–386 (2014). doi:10.1007/s10584-013-0906-1
5. Moss, R. H. R. H. *et al.* The next generation of scenarios for climate change research and assessment. *Nature* **463,** 747–756 (2010).
6. van Vuuren, D. P. *et al.* The representative concentration pathways: An overview. *Clim. Change* **109,** 5–31 (2011).
7. Thomson, A. M. *et al.* RCP4.5: A pathway for stabilization of radiative forcing by 2100. *Clim. Change* **109,** 77–94 (2011).
8. Riahi, K. *et al.* RCP 8.5-A scenario of comparatively high greenhouse gas emissions. *Clim. Change* **109,** 33–57 (2011).
