# Supplementary material for: Ambient PM2.5 exposure and expected premature mortality to 2100 in India under climate change scenarios
Source: Nat Commun. 2018 Jan 22;9:318. doi: 10.1038/s41467-017-02755-y (PMC5778135; doi:10.1038/s41467-017-02755-y)
Supplement: Supplementary file 3 — Description of Additional Supplementary Information [file 41467_2017_2755_MOESM3_ESM.docx]

**Description of Additional Supplementary Files**

File Name: Supplementary Data 1

Description: State level premature mortality burden under RCP scenarios using 5 SSP scenario population distributions. Combination of SSP1 and RCP8.5 is practically impossible. Values are rounded off to nearest 10s.
