# Supplementary material for: Ambient PM2.5 exposure and expected premature mortality to 2100 in India under climate change scenarios
Source: Nat Commun. 2018 Jan 22;9:318. doi: 10.1038/s41467-017-02755-y (PMC5778135; doi:10.1038/s41467-017-02755-y)
Supplement: Supplementary file 4 — Supplementary Data 1 [file 41467_2017_2755_MOESM4_ESM.pdf]

Supplementary Data

State level premature mortality burden under RCP scenarios using 5 SSP scenario population distributions. Combination of SSP1 and RCP8.5 is practically impossible. Values are rounded off to nearest 10s.

SSP1	Baseline Period	RCP 4.5			RCP 8.5		
STATES		**2031-2040**	**2061-2070**	**2091-2100**	**2031-2040**	**2061-2070**	**2091-2100**
**Andaman & Nicobar Islands**	20	10	0	0	10	0	0
**Andhra Pradesh**	22960	15430	7780	2560	16820[#]	11270[#]	5680[#]
**Arunachal Pradesh**	420	400	70	0	480[#]	300[#]	90[#]
**Assam**	20250	16510	9020	3200	16980[#]	11940[#]	6270[#]
**Bihar**	131280	91090	58540	27810	93360[#]	66860[#]	38730[#]
**Chhattisgarh**	17090	12080	6920	2750	12640[#]	8800[#]	4830[#]
**Delhi**	11020	7780	5770	3580	7890[#]	6310[#]	4070[#]
**Goa**	200	140	90	50	140[#]	100[#]	60[#]
**Gujarat**	29810	21370	13990	7180	21610[#]	15370[#]	8890[#]
**Haryana**	16210	11940	8390	4710	11970[#]	8850[#]	5380[#]
**Himachal Pradesh**	3860	2750	1790	900	2730[#]	1990[#]	1150[#]
**Jammu & Kashmir**	6350	5530	3580	1770	5350[#]	3950[#]	2240[#]
**Jharkhand**	31950	22650	14000	6310	23260[#]	16500[#]	9390[#]
**Karnataka**	18820	13690	6430	2080	14750[#]	9470[#]	4420[#]
**Kerala**	8560	5680	2640	980	6040[#]	3720[#]	1660[#]
**Madhya Pradesh**	53990	37950	22980	10320	39380[#]	27760[#]	15460[#]
**Maharashtra**	43580	32080	19000	8430	33070[#]	22910[#]	12430[#]
**Manipur**	410	400	30	0	480[#]	240[#]	50[#]
**Meghalaya**	1320	3520	1850	570	3640[#]	2580[#]	1320[#]
**Mizoram**	250	240	20	0	270[#]	150[#]	40[#]
**Nagaland**	1120	1060	370	50	1150[#]	740[#]	310[#]
**Odisha**	28600	19160	10670	4220	20120[#]	13740[#]	7340[#]
**Punjab**	18700	15120	10770	6090	14910[#]	11250[#]	6920[#]
**Rajasthan**	55300	41350	28130	15330	41530[#]	30180[#]	17940[#]
**Sikkim**	160	170	60	10	190[#]	130[#]	50[#]
**Tamil Nadu**	21650	14430	6220	1670	15890[#]	10140[#]	4580[#]
**Telengana**	18020	12150	6700	2490	12950[#]	8930[#]	4750[#]
**Tripura**	840	1500	760	210	1550[#]	1070[#]	530[#]
**Uttar Pradesh**	236480	159570	106590	55480	162920[#]	117660[#]	69340[#]
**Uttarakhand**	14360	9880	6470	3310	10120[#]	7330[#]	4270[#]
**West Bengal**	69200	49170	30730	13880	50310[#]	35880[#]	20360[#]

SSP2	Baseline Period	RCP 4.5			RCP 8.5		
STATES		**2031-2040**	**2061-2070**	**2091-2100**	**2031-2040**	**2061-2070**	**2091-2100**
**Andaman & Nicobar Islands**	20	10	0	0	10	0	0
**Andhra Pradesh**	22960	17260	9910	3960	18940	14440	8180

**Arunachal Pradesh**	420	440	100	0	540	390	140
**Assam**	20250	18500	11450	4910	19160	15280	8940
**Bihar**	131280	102470	74280	41950	105830	85660	54740
**Chhattisgarh**	17090	13530	8790	4220	14260	11270	6880
**Delhi**	11020	10270	7450	6310	10330	8640	6780
**Goa**	200	160	110	70	160	130	80
**Gujarat**	29810	23890	17680	10830	24330	19600	12550
**Haryana**	16210	13340	10520	6960	13480	11200	7480
**Himachal Pradesh**	3860	3070	2260	1350	3070	2540	1620
**Jammu & Kashmir**	6350	6200	4550	2690	6050	5070	3200
**Jharkhand**	31950	25400	17740	9570	26280	21080	13290
**Karnataka**	18820	15300	8200	3230	16600	12180	6410
**Kerala**	8560	6340	3360	1510	6800	4780	2410
**Madhya Pradesh**	53990	42540	29180	15730	44470	35570	22040
**Maharashtra**	43580	35840	24090	12830	37220	29280	17730
**Manipur**	410	450	30	0	550	310	70
**Meghalaya**	1320	3940	2330	860	4100	3280	1870
**Mizoram**	250	270	30	0	300	190	60
**Nagaland**	1120	1190	480	80	1300	960	450
**Odisha**	28600	21460	13550	6460	22700	17600	10490
**Punjab**	18700	16970	13620	9080	16850	14370	9710
**Rajasthan**	55300	46370	35610	22990	46920	38570	25350
**Sikkim**	160	190	80	10	210	170	80
**Tamil Nadu**	21650	16120	7930	2590	17870	13020	6630
**Telengana**	18020	13590	8510	3840	14580	11430	6800
**Tripura**	840	1680	960	330	1740	1360	760
**Uttar Pradesh**	236480	179220	134860	83110	184360	150320	97600
**Uttarakhand**	14360	11080	8180	4970	11440	9370	6020
**West Bengal**	69200	55070	38860	20960	56770	45750	28730

**SSP3**	**Baseline Period**	**RCP 4.5**			**RCP 8.5**		
STATES		**2031-2040**	**2061-2070**	**2091-2100**	**2031-2040**	**2061-2070**	**2091-2100**
**Andaman & Nicobar Islands**	20	10	0	0	10	0	0
**Andhra Pradesh**	22960	20190	16010	9420	22160	23300	19620
**Arunachal Pradesh**	420	520	160	0	640	640	360
**Assam**	20250	21740	18620	11760	22510	24820	21580
**Bihar**	131280	120940	121240	99730	124900	140090	132220
**Chhattisgarh**	17090	15870	14250	10050	16720	18240	16500
**Delhi**	11020	11700	12690	10500	12070	13900	13320
**Goa**	200	180	180	160	190	200	190
**Gujarat**	29810	27970	28440	25350	28460	31500	29720
**Haryana**	16210	15610	16830	16010	15770	17890	17430
**Himachal Pradesh**	3860	3600	3640	3170	3600	4090	3830

**Jammu & Kashmir**	6350	7300	7410	6400	7110	8240	7710
**Jharkhand**	31950	29850	28760	22640	30880	34180	31790
**Karnataka**	18820	17890	13260	7710	19410	19670	15450
**Kerala**	8560	7420	5430	3570	7940	7720	5800
**Madhya Pradesh**	53990	49950	47350	37370	52200	57680	52920
**Maharashtra**	43580	41910	38790	30190	43500	47050	42130
**Manipur**	410	530	60	0	640	500	180
**Meghalaya**	1320	4650	3840	2100	4840	5380	4610
**Mizoram**	250	320	50	0	360	320	140
**Nagaland**	1120	1390	770	190	1520	1560	1090
**Odisha**	28600	25170	21970	15360	26620	28490	25150
**Punjab**	18700	19980	22110	21310	19830	23300	23110
**Rajasthan**	55300	54520	57770	54180	55130	62550	60590
**Sikkim**	160	220	120	40	250	270	180
**Tamil Nadu**	21650	18840	12820	6180	20890	20980	15910
**Telengana**	18020	15910	13760	9110	17070	18410	16240
**Tripura**	840	1980	1570	800	2050	2220	1860
**Uttar Pradesh**	236480	211100	219140	196050	217150	244510	233860
**Uttarakhand**	14360	13030	13270	11710	13450	15190	14390
**West Bengal**	69200	64680	62850	49480	66650	73940	68490

**SSP4**	**Baseline Period**	**RCP 4.5**			**RCP 8.5**		
STATES		**2031-2040**	**2061-2070**	**2091-2100**	**2031-2040**	**2061-2070**	**2091-2100**
**Andaman & Nicobar Islands**	10	10	0	0	10	10	0
**Andhra Pradesh**	22960	18580	10900	3780	20260	15860	8130
**Arunachal Pradesh**	420	480	110	0	580	430	140
**Assam**	20250	19960	12670	4740	20530	16870	8980
**Bihar**	131280	110990	83050	41310	113890	95670	56140
**Chhattisgarh**	17090	14590	9720	4060	15280	12430	6890
**Delhi**	11020	9860	8750	5010	10370	9340	7320
**Goa**	200	170	130	70	170	140	80
**Gujarat**	29810	25730	19500	10440	26020	21560	12530
**Haryana**	16210	14360	11610	6710	14420	12310	7470
**Himachal Pradesh**	3860	3310	2490	1310	3290	2800	1620
**Jammu & Kashmir**	6350	6750	5240	2890	6550	5800	3540
**Jharkhand**	31950	27420	19650	9250	28180	23320	13360
**Karnataka**	18820	16470	9010	3090	17750	13340	6360
**Kerala**	8560	6830	3690	1440	7260	5240	2390
**Madhya Pradesh**	53990	45890	32280	15170	47660	39270	22110
**Maharashtra**	43580	38580	26510	12300	39790	32120	17630
**Manipur**	410	490	40	0	590	340	70
**Meghalaya**	1320	4250	2590	840	4390	3640	1900
**Mizoram**	250	290	30	0	330	210	60

**Nagaland**	1120	1280	520	80	1390	1050	450
**Odisha**	28600	23140	14970	6220	24320	19400	10490
**Punjab**	18700	18640	16170	10500	18380	17000	11620
**Rajasthan**	55300	50270	40220	23510	50520	43410	26780
**Sikkim**	160	200	80	10	230	190	80
**Tamil Nadu**	21650	17340	8710	2480	19100	14250	6570
**Telengana**	18020	14640	9370	3670	15600	12550	6780
**Tripura**	840	1810	1060	320	1870	1500	770
**Uttar Pradesh**	236480	193760	150070	81110	198050	166960	98940
**Uttarakhand**	14360	11970	9090	4820	12280	10380	6070
**West Bengal**	69200	59440	43110	20400	60870	50640	29060

**SSP5**	**Baseline Period**	**RCP 4.5**			**RCP 8.5**		
STATES		**2031-2040**	**2061-2070**	**2091-2100**	**2031-2040**	**2061-2070**	**2091-2100**
**Andaman & Nicobar Islands**	10	10	0	0	10	0	0
**Andhra Pradesh**	22960	19570	6330	1860	15490	9220	4010
**Arunachal Pradesh**	420	500	60	0	440	250	70
**Assam**	20250	20880	7300	2320	15610	9720	4400
**Bihar**	131280	115040	47340	20160	85680	54300	27090
**Chhattisgarh**	17090	15290	5630	2000	11630	7190	3390
**Delhi**	11020	10340	6300	2870	8040	7330	2890
**Goa**	200	180	70	40	130	80	40
**Gujarat**	29810	27090	11390	5270	19910	12590	6280
**Haryana**	16210	15130	6860	3480	11040	7280	3860
**Himachal Pradesh**	3860	3480	1460	660	2510	1630	810
**Jammu & Kashmir**	6350	6990	2890	1280	4920	3220	1570
**Jharkhand**	31950	28650	11360	4600	21390	13450	6600
**Karnataka**	18820	17360	5230	1510	13590	7750	3110
**Kerala**	8560	7200	2150	710	5570	3040	1170
**Madhya Pradesh**	53990	48040	18650	7500	36230	22650	10860
**Maharashtra**	43580	40670	15490	6170	30480	18780	8810
**Manipur**	410	510	20	0	450	200	30
**Meghalaya**	1320	4430	1480	400	3330	2070	910
**Mizoram**	250	310	20	0	250	120	30
**Nagaland**	1120	1350	300	40	1060	610	220
**Odisha**	28600	24260	8670	3070	18520	11220	5160
**Punjab**	18700	19120	8740	4450	13710	9180	4900
**Rajasthan**	55300	52310	22820	11160	38190	24620	12630
**Sikkim**	160	210	50	10	180	110	40
**Tamil Nadu**	21650	18310	5070	1220	14650	8310	3240
**Telengana**	18020	15400	5450	1820	11920	7300	3350

**Tripura**	840	1890	610	150	1410	860	370
**Uttar Pradesh**	236480	201690	86380	40360	149660	95820	48750
**Uttarakhand**	14360	12490	5250	2410	9310	5980	3000
**West Bengal**	69200	62210	24960	10120	46270	29290	14360
